# Female genital mutilation and cutting: a systematic literature review of health professionals’ knowledge, attitudes and clinical practice

**DOI:** 10.1186/s12914-015-0070-y

**Published:** 2015-12-10

**Authors:** Yvonne Zurynski, Premala Sureshkumar, Amy Phu, Elizabeth Elliott

**Affiliations:** Australian Paediatric Surveillance Unit, The Children’s Hospital at Westmead, Sydney, Australia; Discipline of Paediatrics and Child Health, Sydney Medical School, The University of Sydney, Sydney, Australia; Sydney Children’s Hospitals Network (Westmead), Sydney, Australia

**Keywords:** Female genital mutilation or cutting, Health professionals, Knowledge, Attitudes, Practice

## Abstract

**Background:**

The World Health Organisation (WHO) estimates that 100–140 million girls and women have undergone female genital mutilation or cutting (FGM/C). FGM/C is an ancient cultural practice prevalent in 26 countries in Africa, the Middle East and Asia. With increased immigration, health professionals in high income countries including UK, Europe, North America and Australia care for women and girls with FGM/C. FGM/C is relevant to paediatric practice as it is usually performed in children, however, health professionals’ knowledge, clinical practice, and attitudes to FGM/C have not been systematically described. We aimed to conduct a systematic review of the literature to address this gap.

**Methods:**

The review was conducted according to guidelines of the Preferred Reporting Items for Systematic Reviews and Meta-Analyses (PRISMA) statement and registered with the PROSPERO International Prospective Register of Systematic Reviews (CRD42015015540, http://www.crd.york.ac.uk/PROSPERO/). Articles published in English 2000–2014 which used quantitative methods were reviewed.

**Results:**

Of 159 unique articles, 18 met inclusion criteria. The methodological quality was poor - six studies met seven of the eight quality criteria. Study participants included mainly obstetricians, gynaecologists and midwives (15 studies). We found no papers that studied paediatricians specifically, but two papers reported on subgroups of paediatricians within a mixed sample of health professionals. The 18 articles covered 13 different countries: eight from Africa and 10 from high income countries. Most health professionals were aware of the practice of FGM/C, but few correctly identified the four FGM/C categories defined by WHO. Knowledge about FGM/C legislation varied: 25 % of professionals in a Sudanese study, 46 % of Belgian labour ward staff and 94 % of health professionals from the UK knew that FGM/C was illegal in their country. Health professionals from high income countries had cared for women or girls with FGM/C. The need to report children with FGM/C, or at risk of FGM/C, to child protection authorities was mentioned by only two studies.

**Conclusion:**

Further research is needed to determine health professionals’ attitudes, knowledge and practice to support the development of educational materials and policy to raise awareness and to prevent this harmful practice.

## Background

The World Health Organisation (WHO) estimates that between 100–140 million girls and women have undergone female genital mutilation or cutting (FGM/C) [1]. FGM/C is usually performed in children aged between 1 month and 15 years, and is therefore relevant to paediatric practice [2]. There are different types of FGM/C procedures ranging from “nicking” or “pricking” the prepuce, to complete removal of the clitoris or infibulation, when the vaginal opening is narrowed by cutting and repositioning the inner or outer, labia, with or without removal of the clitoris [1, 3]. FGM/C is an ancient cultural practice, predating both the Bible and the Koran and has no basis in religion [4]. FGM/C is currently customary in over 26 countries in Africa, the Middle East and Asia, with a prevalence of 70 % or more reported in 11 African countries including Somalia, Egypt, Sierra Leone, Sudan, Mali, Eritrea, and Ethiopia [2]. There are no medical or health indications for FGM/C. FGM/C is harmful and immediate complications include bleeding, pain, infections and significant psychological trauma [1, 2, 5, 6]. Long term complications include recurrent urinary infections, birthing difficulties including need for emergency caesarean section, third-degree vaginal tears, and ongoing psychological and sexual problems [1, 2, 4–8].

All forms of FGM/C whether performed by medical practitioners or other “cultural practitioners” are illegal in at least 20 countries in Africa including Kenya, Nigeria and Egypt [9], and in high income countries such as Australia, New Zealand, United Kingdom, Republic of Ireland, Canada, many European Countries, and 15 of the 52 States of the USA have law where parents/guardians and circumcisers are subject to prosecution [4–6, 10–12]. Furthermore, it is illegal to organise for FGM/C procedures to be performed overseas in children resident in many of these high income countries [5–7, 10, 12]. FGM/C is a child protection issue and in many countries, mandatory reporting to authorities is required by health professionals who identify children who have undergone FGM/C or who are believed to be at risk of FGM/C [4–7, 10–12]. FGM/C violates the UN Charter of Human Rights, the UN Charter of Women’s Rights, the Charter of the Rights of the Child, and the Charter of Rights of the African Child [13–16].

Medicalization of FGM/C refers to the procedure being performed in a medical setting, often by a doctor [17, 18]. A recent study from the UK reported that of 27 girls who had FGM/C, it was known to have been performed by a doctor in a medical setting in 71 % [19]. Medicalization is often supported by those who practice FGM/C because they believe it offers “harm reduction” by preventing immediate medical complications [17, 18]. However, the involvement of healthcare providers in FGM/C in any setting has been condemned by the WHO because it does not prevent long-term medical or psychological complications and legitimises continuation of FGM/C in some communities [1, 3].

Many women with FGM/C and girls at risk of FGM/C are now living in the UK, Europe, North America, Australia and New Zealand due to the increasing immigration from countries where FGM/C is prevalent [4–7, 10–12]. The prevalence of FGM/C in girls and women living in these countries is unknown, because procedures tend to be organised by families in private, often outside the mainstream health system, and information about FGM/C is not routinely collected or coded in medical records. Furthermore, girls may be taken for FGM/C to the family’s country of origin [5]. Thus, FGM/C may only become apparent to health professionals when girls or young women present with complications, or when women need obstetric and gynaecological care [5, 7, 20].

As the immigrant communities in high income countries become larger and increasingly multicultural and ethnically diverse, health professionals are more likely to see women and girls with FGM/C or at risk of FGM/C, in their clinical practice. In this systematic review of the literature we aimed to identify, describe and analyse publications reporting the knowledge, attitudes and clinical practices related to FGM/C among health professionals internationally. We aimed to answer the following questions:

1. Do health professionals have experience of FGM/C in their clinical practice?
2. Do health professionals have adequate knowledge about FGM/C categories, complications, and high risk groups and do they have access to education and training opportunities?
3. Do health professionals have adequate knowledge about laws relating to FGM/C?
4. What are the attitudes and beliefs of health professionals towards the practice of FGM/C?

## Methods

Systematic review of the literature using the terms “female genital mutilation”, “female genital cutting” or “female circumcision” combined with MESH terms: “Paediatrics”, “Child Health” and keywords: “paediatrician”, “practice guidelines,” “attitudes” “knowledge” and “education” was conducted. Databases including MEDLINE, CINHAL and SCOPUS were searched applying limits: year of publication 2000–2014; human; English language.

The review was conducted according to guidelines of the Preferred Reporting Items for Systematic Reviews and Meta-Analyses (PRISMA) statement and registered with the PROSPERO International Prospective Registerof Systematic Reviews (CRD42015015540, http://www.crd.york.ac.uk/PROSPERO/).

The titles and abstracts of all articles identified through the literature search were scanned for relevance. Documents were selected for full review if they specifically mentioned FGM/C, and reported primary data on health professionals’ knowledge attitudes and clinical practice related to FGM/C.

### Definitions

*WHO definitions of the 4 types of FGM/C*:

1. *Clitoridectomy*: partial or total removal of the clitoris (a small, sensitive and erectile part of the female genitals) and, in very rare cases, only the prepuce (the fold of skin surrounding the clitoris).
2. *Excision*: partial or total removal of the clitoris and the labia minora, with or without excision of the labia majora (the labia are “the lips” that surround the vagina).
3. *Infibulation*: narrowing of the vaginal opening through the creation of a covering seal. The seal is formed by cutting and repositioning the inner, or outer, labia, with or without removal of the clitoris.
4. *Other*: all other harmful procedures to the female genitalia for non-medical purposes, e.g. pricking, piercing, incising, scraping and cauterizing the genital area.
  Other definitions:
5. *De-infibulation*: is the surgical procedure to open up the closed vagina of FGM type 3 and is often performed on the wedding night, and prior to childbirth.
6. *Reinfibulation*: The re-stitching of FGM type III to reclose the vagina after childbirth.

### Inclusion criteria

#### Design

Human observational studies, including cross sectional, cohort or population-based studies that used quantitative methodology.

#### Participants

Health professionals including paediatricians, obstetricians, gynaecologists, family doctors, nurses, midwives or students of medicine, nursing, midwifery or other health disciplines.

#### Outcomes

Measures of knowledge about FGM/C, attitudes/beliefs towards FGM/C and experience of FGM/C in clinical practice.

#### Exclusion criteria

- Publications reporting patient or community knowledge or attitudes
- Publications that used qualitative study designs
- Publications reporting on genital cosmetic procedures
- Foreign language publications

### Quality assessment

Publications were assessed and scored for representativeness and survey tool validity. Quality measures included: sample description (1 point for each detail provided: profession, age, gender of respondents and response rate); sampling method (description of site/setting – 1 point, sampling procedure described - 1 point); and survey validity (1 point if survey pre-tested and 1 point if the survey was reviewed by content experts), for a maximum score of eight points.

### Data extraction and analysis

Data were extracted by two researchers independently (YZ, AP). Any inconsistencies were resolved by checking full-text versions of the documents and discussion with the review team. All proportions reported in the original documents have been rounded up to whole percentages for ease of reading and interpretation.

## Results

**Fig. 1:**
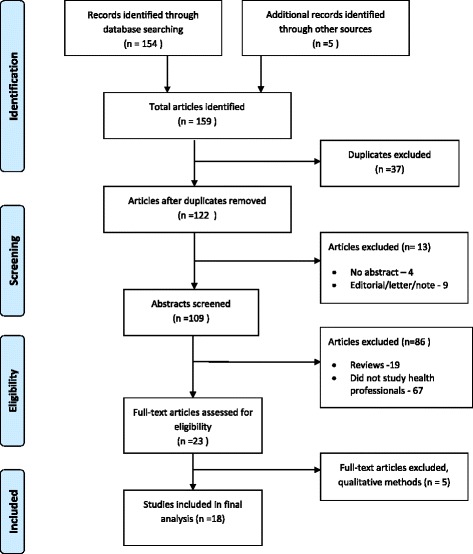
Identification and selection of studies for review

One hundred and fifty nine potentially relevant articles were identified. After exclusion of duplicates there remained 122 unique publications. Editorials, letters, notes and publications that did not have abstracts (mainly opinion pieces) were excluded, leaving 109 abstracts for screening. Of the 109 abstracts screened, 67 did not study health professionals and 19 were reviews which did not include primary data. Twenty-three full text articles were reviewed in detail and 5 of these were excluded because they used qualitative methods, leaving 18 articles for analysis (Fig. 1) [20–37].

Of the 18 publications, eight originated from low-middle income countries in Africa, mainly from Nigeria and Egypt (Table 1). Ten came from high income countries: five from Europe, three from the UK, one from Australia/New Zealand (ANZ), and one from the USA (Table 1). We found no studies that specifically focussed on paediatricians. Four studies reported on mixed samples, which included paediatricians, but only two of these analysed paediatricians as a separate subgroup (Table 1). Seventeen studies reported on health professionals’ knowledge, 13 on practice and 12 on attitudes, with only four studies from high income countries reporting on health professionals’ attitudes (Table 1).

**Table 1:** Characteristics of studies included in the review

Reference	Country	Study design and method	Domains assessed			Sample	N	Response rate
			Attitudes	Knowledge	Practice			
Publications from African Countries								
Ashimi et al. 2014 [21]	Nigeria	Cross-sectional; self- administered survey	Yes	Yes	No	Nurses	350	84 %
Kaplan et al. 2013 [22]	Gambia	Cross-sectional; survey administered face to face	Yes	Yes	Yes	Nurses, community nurses and midwives	468	NR
Ali et al. 2012 [23]	Sudan	Survey administered via face to face interview	Yes	Yes	Yes	Midwives *(~63 % of midwives were illiterate)*	157	NR
Dike et al. 2012 [24]	Nigeria	Cross-sectional survey	Yes	Yes	No	Student nurses and midwives	269	95.7 %
Rasheed et al. 2011 [25]	Egypt	Cross sectional; self- administered survey	Yes	No	Yes	^a^Nurses; junior and senior physicians		
Refaat 2009 [26]	Egypt	Cross-sectional Survey	Yes	Yes	Yes	^a^Physicians	193	68 %
Mostafa et al. 2006 [27]	Egypt	Random sample; Survey	Yes	Yes	No	5^th^ year medical students	330	90.3 %
Onuh et al. 2006 [28]	Nigeria	Cross-sectional; Survey	Yes	Yes	Yes	Nurses practising in a hospital	182	94.3 %
Publications from “Western Countries”								
Caroppo et al. 2014 [29]	Italy	Purposive sample; Self-administered survey	No	Yes	Yes	Physicians, social workers, psychologists, “health assistants” working in an asylum seeker centre	41	100 %
Purchase et al. 2013 [30]	UK	Cross-sectional; survey	No	Yes	No	Obstetricians and Gynaecologists	607	20.1 %
Relph et al. 2013 [31]	UK	Cross-sectional; Survey	Yes	Yes	No	Health care professionals	79	92.9 %
Moeed et al. 2012 [20]	Australia and New Zealand	Cross- sectional; Survey	No	Yes	Yes	Obstetricians and Gynaecologists and trainees	564	18.5 %
						FGM/C workers	34	91.9 %
Hess et al. 2010 [32]	USA	Randomised Survey	Yes	Yes	Yes	Nurse-midwives	243	40.3 %
Kaplan-Marcusan et al. 2009 [33]	Spain	Cross-sectional; Survey at two time points (2001 and 2004)	Yes	Yes	Yes	^b^Primary health care professionals	280 (2001)	80 % (2001)
							296 (2004)	62 % (2004)
Leye 2008 [34]	Belgium	Cross-sectional; Survey	Yes	Yes	Yes	Gynaecologists and trainees	333	46 %
Zaidi et al. 2007 [35]	UK	Cross-sectional; Survey	No	Yes	Yes	Labour ward staff	45	100 %
Tamaddon et al. 2006 [36]	Sweden	Cross-sectional; Survey	No	Yes	Yes	^b^Health professionals	796	28 %
Jager et al. 2002 [37]	Switzerland	Cross-sectional; Survey	No	Yes	Yes	Obstetricians and gynaecologists	454	39.1 %

^a^Sample included paediatricians but did not report on paediatricians separately;

^b^Sample included paediatricians and paediatricians were compared with other professionals;

*NA* Not applicable

*NR* Not Reported

### Quality assessment

Publications were scored according to our pre-determined quality assessment matrix (Table 2). Only one publication scored the maximum eight points. Twelve (67 %) papers described the age of the participants and 11(61 %) reported gender. A description of the setting was lacking in two studies, sampling procedures were not described in three. (Table 2). Six (33 %) of the surveys were pre-tested, five (22 %) were reviewed by content experts, and two (11 %) were both pre-tested and reviewed by a content expert. Nine studies did not report any survey validation. Most of the studies are unlikely to be representative. Three studies from high income countries were set in specialist facilities serving migrant communities in which FGM/C is common and the health professionals surveyed had frequent experience with women affected by FGM/C.[29, 31, 35] Two studies did not report a response rate and in 5 studies the response rate was <50 %, (Table 1).

**Table 2:** Assessment of methodological quality of studies included in the review

Reference	Representativeness						Survey validity		Score out of 8
	Profession of respondents described	Age or years of practice	Gender	Setting	Sampling procedure	Response rate reported	Pre-test	Expert review	
Publications from African Countries									
Ashimi et al. 2014 [21]	Yes	Yes	Yes	Yes	Yes	Yes	Yes	No	7
Kaplan et al. 2013 [22]	Yes	Yes	Yes	Yes	No	No	Yes	Yes	7
Ali et al. 2012 [23]	Yes	Yes	No^a^	Yes	No	No	No	No	3
Dike et al. 2012 [24]	Yes	Yes	Yes	Yes	Yes	Yes	Yes	No	7
Rasheed et al. 2011 [25]	Yes	No	No	Yes	No	Yes	No	No	3
Refaat 2009 [26]	Yes	Yes	Yes	No	Yes	Yes	No	No	5
Mostafa et al. 2006 [27]	Yes	Yes	Yes	Yes	Yes	Yes	No	No	6
Onuh et al. 2006 [28]	Yes	Yes	Yes	Yes	Yes	Yes	Yes	No	7
Publications from “Western Countries”									
Caroppo et al. 2014 [29]	Yes	No	Yes	Yes	Yes	Yes	No	No	5
Purchase et al. 2013 [30]	Yes	Yes	No	Yes	Yes	Yes	No	No	5
Relph et al. 2013 [31]	Yes	Yes	Yes	Yes	Yes	Yes	Yes	No	7
Moeed et al. 2012 [20]	Yes	No	No	No	Yes	Yes	No	No	3
Hess et al. 2010 [32]	Yes	Yes	Yes	Yes	Yes	Yes	No	Yes	7
Kaplan-Marcusan et al. 2009 [33]	Yes	Yes	Yes	Yes	Yes	Yes	No	No	6
Leye 2008 [34]	Yes	Yes	Yes	Yes	Yes	Yes	Yes	Yes	8
Zaidi et al. 2007 [35]	Yes	No	No	Yes	Yes	Yes	No	Yes	5
Tamaddon et al. 2006 [36]	Yes	No	No	Yes	Yes	Yes	No	Yes	5
Jager et al. 2002 [37]	Yes	No	No	Yes	Yes	Yes	No	No	4

“Yes” indicates that this criterion was adequately reported in the paper

^a^The sample consisted of “midwives” and it is assumed that all would have been female given the cultural setting for this study

1. Do health professionals have experience with FGM/C in their clinical practice?

Five surveys in high income countries reported that health professionals who responded provided care to women with FGM/C, including 75.3 % of obstetricians/gynaecologists in ANZ [20]; 40 % of nurse-midwives in the USA [32]; 50 % of Swiss obstetricians/gynaecologists [37]; 60 % of Swedish health providers including paediatricians [36]; 12 % of paediatricians, 80 % of gynaecologists responding to a Spanish survey [33]; and 58 % of Belgian gynaecologists [34], ( Table 3). Despite working in an asylum seeker health service in Italy, which serves refugees from high prevalence countries, 71 % of health professionals reported that they had never met or assisted a woman with FGM/C [29].

**Table 3:** Reported experience of FGMC in clinical practice

Reference	Country	Had seen patients with FGMC	Managed women or girls with FGMC/FGMC complications; used prevention measures	Has performed FGMC or has been asked to perform FGMC	Clinical Guidelines/Clinical Education to support practice
Publications from African Countries					
Kaplan et al. 2013 [22]	Gambia	41 % - had seen a girl with complications of FGM/C	41% - had seen a girl with complications of FGM/C	8 % - had performed FGM/C	NR^a^
				69 % - FGM/C is practiced in my family/household	
Ali et al. 2012 [23]	Sudan	NR	NR	81 % had performed FGM/C during their career	NR
				Each of these midwives had performed 5–88 FGM/C procedures in the previous year	
Rasheed et al. 2011 [25]	Egypt	NR	NR	None of the nurses had performed FGM/C	NR
Refaat 2009 [26]	Egypt	NR	NR	19 % - performed FGM/C	NR
				34 % of those who perform FGM/C reported complications among patients	
Onuh et al. 2006 [28]	Nigeria	NR	NR	7 % - currently practice FGM/C	NR
				14 % have practiced FGM/C in the past	
				58 % - will perform FGM/C in the future if compelled to do so	
Publications from “Western Countries”					
Caroppo et al. 2014 [29]	Italy	71 % - never met or assisted a woman with FGM/C despite working in an asylum seeker facility	76 % - stated they would refer the woman for care elsewhere, with many different options provided		34 % were aware of guidelines/procedures for the management of women with FGM/C
Purchase et al. 2013 [30]	UK	87 % - had been involved in the care of a girl/woman with FGM/C		3 midwives had been asked to perform FGM/C in a child or to re-infibulate after delivery	26 % - had sufficient training in FGMC
		20 % - had seen >10 cases			31 % - reported that the hospital/trust had screening for FGM/C procedures
					21 % - there was an FGM/C specialist (obstetrician or midwife) at the hospital trust
					40 % - had training in de-infibulation
Relph et al. 2013 [31]	UK	59 % had been involved in the care of a woman with FGM/C	NR	NR	NR
Moeed et al. 2012 [20]	Australia and New Zealand	76 % see women from African countries and from the Middle East	47 % had seen at least one woman or girl with complications related to FGMC – “most commonly” urinary problems; problems in labour and dyspareunia	21 % - of O&G specialists asked to re-infibulate after birth	NR
		75 % saw at least one woman with FGM/C in the last 5 years	“A few” reported psychosexual complications	12 % - of those who had been asked had done so:	
		Most saw 1–5 women with FGMC in the last 5 years		38 % of the FGM/C workers had heard of re-suturing taking place; one respondent indicated that re-suturing had taken place >50 times	
				2 (0.5 %) respondents had been asked to perform FGM/C on a baby, young girl or woman	
				One was asked on 1–5 occasions; the other 6–10 occasions	
				1 % of the O&G specialists had convincing evidence that the procedure was done in Australia or NZ	
				10 % of the FGM/C workers were aware of convincing evidence that the procedure was being performed in Australia or NZ	
Hess et al. 2010 [32]	USA	43 % - of certified nurse- midwives had seen women with FGM/C in their practice	Problems associated with FGMC not discussed consistently		NR
			20 % discussed circumcision of daughters, nieces, grand- daughters “Often” or “Always”		
			78 % never discussed infertility		
Kaplan-Marcusan et al. 2009 [33]	Spain	2001	NR	91 % of paediatricians had an interest in FGM/C	NR
		6 % - of all HP surveyed had seen cases in practice		42 % of paediatricians were aware of guidelines and protocols	
		7 % - of paediatricians saw FGM/C			
		2004			
		16 % - had seen FGMC in practice			
		19 % – of paediatricians saw FGM/C			
		FGM/C was seen by females more often than males			
Leye 2008 [34]	Belgium	58 % had seen women or girls with FGM/C in their practice	Consulted regarding complications:	2 % [6] respondents had been asked to perform FGM/C in Belgium	
		Most common forms:	1 % - acute complications	4 % [13] had been asked whether FGMC could be performed in Belgium	51 % wanted guidelines on FGM/C
		56 – infibulation	1 % - fertility problems	9.5 % [31] gynaecologists had heard that FGM/C had been performed in Belgium	45 % sought more information about FGM/C after seeing patients with FGM/C
		40 – Excision	2 % - psychological problems		
		3 – sunna^b^	4 % - fistulae		
		7 patients , 14 years old	15 % - pregnancy and delivery problems		
		23 patients 15–18 years old	18 % - chronic pain		
		The rest were 19 years or older	19 % - urinary tract infections		
		Patients were from: Somalia, Ethiopia, and other including Nigeria, Egypt, Mali, Senegal	41 % - sexual dysfunction		
			35 % - of those looking after pregnant women tried to persuade the mother not to perform FGMC if the child was a daughter		
			65 % - said they would not do any prevention		
Zaidi et al. 2007 [35]	UK	80 % had seen women with FGM/C in their practice	NR	NR	NR
Tamaddon et al. 2006 [36]	Sweden	60 % had seen at least one patient with FGM/C	39 % - had seen patients with long-term complications of FGM/C	5 % - had been asked about performing FGM/C in Sweden; 4 of these were paediatricians	NR
			1 % - had seen patients with complications due to recently performed FGC	10 % - had been asked to perform reinfibulation after birth	
			2 of these 7 were paediatricians, 4 midwives, 1 gyneacologist		
Jager et al. 2002 [37]	Switzerland	51 % - had seen women with FGM/C in their practice in Switzerland	NR	21 % - had been asked to re-infibulated after birth	FGM/C is not included in the undergraduate medical curriculum
		73 % - from the French-speaking region of Switzerland had seen women with FGM/C in their practice		2 gyneacologists have been asked to perform FGM/C in young girls	There is no reporting system for FGM/C
				4 gyneacologists were asked where FGMC could be performed in Switzerland	
				12 gyneacologists said that they knew of FGM/C being performed in Switzerland	

^a^NR = Not reported; ^b^Sunna- Equivalent to the WHO Type 1 – cliteridectomy

Some obstetricians, gynaecologists and midwives working in high income countries had been asked to re-infibulate women after delivery and some had done so (Table 4). Four studies reported that health professionals in high income countries had been asked to perform FGM/C in babies or young girls, or to provide information about where to get FGM/C procedures done: two respondents to the ANZ survey [20]; 6 respondents to the Belgian study [34]; two respondents to the Swiss survey [37] and seven health professionals including two paediatricians in a Swedish survey [36] (Table 3).

**Table 4:** Health professionals’ reported knowledge about FGMC

Reference	Country	Knowledge of FGM/C ; FGM/C types ; high risk groups	Knowledge about complications	Knowledge about legislation / clinical guidelines
Publications from African Countries				
Ashimi et al. 2014 [21]	Nigeria	91 % - had heard of FGM/C	77 % - haemorrhage	NR^a^
		40 % - did not know any of the 4 types	73 % - transmission of infectious disease (HIV, hepatitis and tetanus)	
		49 % identified “Angurya and Gishiri”^b^ as forms of FGM/C	63 % - sexual dysfunction	
			54 % - difficult birth	
			48 % - epidermal cysts	
Kaplan et al. 2013 [22]	Gambia	NR	53 % - haemorrhage	NR
			59 % - transmission of infectious disease	
			46 % - difficult birth	
			25 % - sexual dysfunction	
			21 % - affects health and welfare of women and girls	
Ali et al. 2012 [23]	Sudan	7 % - identified all 4 types correctly	46 % - transmission of infectious disease (HIV)	25.5 % - FGM/C is illegal
		545 % - identified type 1 correctly	64 % - sexual dysfunction	74.5 % - FGM/C is legal
			29 % - infertility	
Dike et al. 2012 [24]	Nigeria	NR	86 % - haemorrhage	100 % - FGM/C is banned in some states
			84 % - transmission of infectious disease (HIV)	96 % - FGM/C is a crime against humanity
			27 % - difficult birth	
			7 % - sexual dysfunction	
Rasheed et al. 2011 [25]	Egypt	NR	66 % - knew about complications of FGM/C	NR
Refaat 2009 [26]	Egypt	76 % - know the type usually performed in Egypt (type II)	75 % - haemorrhage	NR
			70 % - sexual dysfunction	
			64 % - shock	
			63 % - genital disfigurement	
			14 % - NO complications (if done by a physician or gynaecologist)	
Mostafa et al. 2006 [27]	Egypt	52 % - correctly identified type I	62 % - aware that FGMC can cause complications including:	17 % - knew Egyptian law which states that FGM/C cannot be performed by a non-physician
		30 % - identified type II	48 % - short-term physical	28 % - reported that FGM/C violates the medical ethical principles of “do no harm” and “no not kill”
		5 % - identified type III	39 % - long term physical	
			62 % - psychosocial complications	
			59 % - sexual dysfunction	
Onuh et al. 2006 [28]	Nigeria	100 % - identified at least one type of FGMC	98 % - haemorrhage	NR
		38 % - identified Type I and Type II ONLY as FGM/C	81 % - transmission of infectious disease	
		7 % - identified all 4 types correctly	54 % - transmission of HIV	
			80 % - difficult birth	
			55 % - scars and keloid formation	
			21 % - infertility	
			59 % - sexual dysfunction	
Publications from “Western Countries”				
Caroppo et al. 2014 [29]	Italy	9 % - knew that there are different types of FGM/C depending on the woman’s country of origin	5 % - knew how to manage a woman with FGMC	44 % - knew that Italy has a law prohibiting FGMC practice
Purchase et al. 2013 [30]	UK	NR	92 % - identified each of the long term complications	94 % - FGM/C always illegal in the UK
			75 % - HIV/hepatitis risk	79 % - were aware of the FGM/C Act
			74 % - pelvic infection	84 % - knew to contact a child protection officer if they thought a child was at risk
			10 % - associated psychiatric syndromes	
			To prevent complications during labour:	
			74 % - knew that defibulation should take place pre-conception	
			31 % - knew that defibulation is recommended at ~ 20 weeks pregnancy	
			52 % - unaware of referral pathways	
Relph et al. 2013 [31]	UK	100 % - aware of the practice of FGM/C	76 % - haemorrahge	72 % - aware of UK legislation on FGM/C
		58 % - knew there are 4 types of FGM/C	32 % - knew that defibulation should be performed before pregnancy to avoid complications	89 % - family/religious figure performing FGM/C in UK is illegal
		93 % of senior doctors		77 % - UK doctor performing FGM/C in UK is illegal
		50 % of junior doctors		67 % - reinfibulation after delivery is illegal
		40 % - confident in diagnosing FGM/C		78 % - sending a child abroad for FGM/C is illegal
Hess et al. 2010 [32]	USA	18 % - knew that both Muslim and Christian women may have FGM/C	71 % - of nurse midwives who did not have direct experience with FGMC knew about FGMC complications , compared with 89 % of those who had direct experience	56 % - knew that it is illegal to perform FGM/C in girls and young women aged <18 years
		39 % - knew FGM/C is NOT required by either religion	Over a half of respondents did not know that circumcised women avoid health care due to stigma and legal implications	
		Nurse midwives with direct practice experience of FGM/C scored better on a knowledge test		
Kaplan-Marcusan et al. 2009 [33]	Spain	97 % knew what FGM/C is	NR	20 % - aware of protocols or guidelines
		Able to identify the 4 types:		42 % - of paediatricians aware of protocols or guidelines
		41 % - of all professionals		
		68 % - of O&G		
		55 % - of paediatricians		
		38 % - general medicine		
		79 % - said they knew high risk countries		
		22 % - actually able to identify the high risk countries		
Leye 2008 [34]	Belgium	NR	NR	46 % - knew that FGM/C was illegal in Belgium
				24 % - knew which types of FGM/C were included under the law
				1 % (4 respondents) - knew of guidelines and information about FGM/C in their hospital
Zaidi et al. 2007 [35]	UK	98 % - knew what FGMC was	84 % - knew of complications associated with FGMC	40 % - knew the details of the UK FGM/C Act
		42 % - knew that there were different types of FGMC	70 % - knew that the best time for defibulation was before pregnancy (if FGMC diagnosed before pregnancy)	
		4 % - correctly classified the 4 types	80 % - knew that defibulation should be done during pregnancy if diagnosed during pregnancy	
		84 % - knew the high risk groups	54 % - knew that an anterior episiotomy should be performed if the woman is in the 2^nd^ stage of labour	
		58 % - were NOT aware that women at risk should be identified during antenatal visits		
Tamaddon et al. 2006 [36]	Sweden	28 % - said they had adequate knowledge about FGM/C	NR	NR
		20 % - of paediatricians said they had adequate knowledge about FGM/C		
Jager et al. 2002 [37]	Switzerland	NR	NR	Representatives from the Departments of Health in each Canton, did not know of any guidelines on FGM/C in their Canton

^a^NR = Not reported ^b^ *Angurya*: is a form of FGMC type 4 that involves the scraping of tissue around the vaginal opening. *Gishiri*: is a form of FGMC type 4 where a long knife is inserted into the vagina and backward cuts from the vagina's anterior wall into the perineum are made

Survey respondents in high income countries reported that they knew that FGM/C was being practised in children including in Belgium and Switzerland [34, 37]. Approximately 20 % of obstetricians/gynaecologists responding to the ANZ survey believed that women presenting to them with FGM/C probably had the procedure done in Australia or New Zealand [20].

Five surveys of health professionals in Nigeria [28], Egypt [25, 26], Gambia [22] and the Sudan [23] reported on whether the respondents had performed or had been asked to perform FGM/C procedures (Table 3). The study of Sudanese midwives reported that 81 % of respondents had performed FGM/C multiple times [23]. In contrast, among nurses and community midwives surveyed in Gambia, only 7.6 % had performed the procedure but 68.6 % said that FGM/C was practiced in their household or family [22]. Among nurses surveyed in Nigeria, 7 % currently practiced FGM, 14 % had practiced in the past and 58 % said they would perform FGM/C if required [24]. None of the nurses surveyed in Egypt [25] had performed FGM/C, but 19.2 % of Egyptian doctors surveyed had performed FGM/C and of these 24 % reported complications due to FGM/C [26].

2. Do health professionals have adequate knowledge about FGM/C types, complications, high risk groups and do they have access to education and training opportunities?

Knowledge about the FGM/C types varied widely; few health professionals in high income countries knew that there were 4 different types of FGM/C and fewer were able to identify the 4 types (Table 4). The Spanish study was an exception with 85 % of O&G and 55 % of paediatricians able to identify the 4 types of FGM/C [33]. Knowledge of the 4 types of FGM/C was also poor among respondents surveyed in Africa, however, most respondents knew of the type of FGM most commonly practised in their local area e.g. 76 % of Egyptian health professionals knew of type II FGM/C which is usually performed in Egypt [26].

In a study in North East London, 50 % of senior doctors and only 7 % of junior doctors had formal training in FGM/C; midwives were more confident in diagnosing FGM/C than doctors and 75 % of medical students were aware of FGM/C complications [31]. However, in an earlier study of midwives and doctors who attend births, also in London, only 4 % could correctly identify the different types of FGM/C and knowledge about the correct procedures to de-infibulate women during labour was poor for ~45 % of the respondents [35].

Survey respondents correctly identified a number of short and long-term complications of FGM/C although some studies reported that respondents knew of no complications after FGM/C (Table 4). Almost all participants (92 %) in the study in Birmingham, UK, correctly identified most long-term complications of FGM/C except for HIV/hepatitis and pelvic infection [30]. Only two studies asked about knowledge of psychological or psychosocial complications after FGM/C [30, 31].

Eleven per cent of Belgian doctors aged less than 40 years had been taught about FGM/C but only 1 % knew of guidelines or information about FGM/C in their hospital [34]. Education on FGM/C is not regularly included in undergraduate education in Switzerland [37]. Few Swedish paediatricians knew about FGM/C and the motives behind FGM/C [36], and Norwegian health professionals felt that they had inadequate knowledge and skills about FGM/C and they called for specific training in how to speak with women and families about FGM/C and which words to use when raising the issue (Table 4).

In a survey of obstetricians and other health professionals working in a large UK clinic, 26 % believed they had adequate training in FGM/C, 41 % had been trained in de-infibulation, 31 % knew that the hospital regularly screened for FGM/C and that the hospital had an obstetrician and a midwife that specialised in FGM/C [30]. Among paediatricians surveyed in Spain, 42.3 % were aware of protocols and guidelines about FGM/C [33]. In the study from Belgium, 51 % of gynaecologists surveyed, wanted relevant guidelines on FGM/C, 35 % said they tried to prevent mothers who had FGM/C from allowing FGM/C to be performed in their female children, but 65 % said they would not do any prevention [34].

3. Do health professionals have adequate knowledge about laws related to FGM/C?

In a recent study of members (*N* = 607) of the Royal College of Obstetricians and Gynaecologists in the UK, 94 % understood that FGM/C is always illegal in the UK but 21 % were unaware of the FGM/C Act, (Table 4) [30]. The majority (84 %) of respondents said they would speak with a child protection officer if they suspected a child was at risk of FGM/C [30]. In the London study by Zaidi et al. 40 % of health professionals were familiar with the FGM/C Act [35]. Relph et al. reported that only 60 % of the UK health professionals surveyed were aware of current UK FGM/C law [31]. In the Belgian survey of gynaecologists, 45.5 % knew that FGM/C was illegal in Belgium, the majority (85.6 %) understood that FGM/C constituted violence against women, but only 60 % felt that it violated human rights [34]. Over a half (56 %) of midwives surveyed in a USA study knew that FGM/C was against the law [32]. In the Italian study of health professionals working with asylum seekers from FGM/C prevalent countries, less than half knew about the law prohibiting FGM/C in Italy [29].

Only 25 % of the Sudanese respondents [23] and 17 % of Egyptian respondents [24] knew that FGM/C was illegal in their country (Table 4). Furthermore, 35 % of Egyptian doctors responding to survey conducted by Refaat et. al. did not approve of the law banning FGM/C [26]. However, all participants surveyed in a Nigerian study knew that FGM/C was illegal in some states [24].

4. What are the attitudes and beliefs of health professionals towards the practice of FGM/C?

Beliefs about the reasons for performing FGM/C varied widely with some respondents from both high income countries and from African countries believing that FGM/C was done for religious reasons (Table 5). Surveys from African countries also cited other reasons including cultural, social, medical economic and cosmetic, included “preservation of virginity”, “curbing promiscuity”, and “improving the appearance of genitalia,” while those from high income countries only cited cultural/traditional reasons or religious reasons (Table 5). In four surveys, between 4 % and 48 % of health professionals indicated that they would agree for their own daughters to undergo FGM/C [21, 25, 27, 28].

**Table 5:** Health professionals’ attitudes towards FGMC

Reference	Country	Beliefs about the reasons for performing FGM/C	Support for and intentions for performing FGM/C	Beliefs and attitudes about the law and educational needs
Publications from African Countries				
Ashimi et al. 2014 [21]	Nigeria	53 % - prevent promiscuity	4 % would support FGM/C	NR^a^
		28 % - preserve virginity	4 % would perform FGM/C	
		16 % - socio-cultural acceptance	4 % of respondents (all women) would allow daughters to undergo FGM/C	
		10 % - religious reasons		
		8 % - medically beneficial		
Kaplan et al. 2013 [22]	Gambia	54 % - mandatory religious practice	43 % - were supportive of the continuation of FGM/C practice	NR
		48 % - cultural practice	47 % - intended to subject their daughters to FGM/C	
		14 % - preserve virginity	43 % - medicalising FGMC would make the practice safer	
		1 % - it does not violate human rights	73 % - Health care workers have a role in eliminating FGMC	
			55 % – FGM/C cannot be eliminated in The Gambia	
			78 % - men should be involved in the debate about FGM/C	
			13 % - girls that have not undergone FGM/C should be discriminated against	
Ali et al. 2012 [23]	Sudan	51.2 % - cultural	19 % - all forms of FGM/C are harmful	NR
		26 % - religious	76 % - only some forms are harmful	
		23 % - economic	5 % - all forms are not harmful	
Dike et al. 2012 [24]	Nigeria	51 % - prevent promiscuity	100 % would NOT have their daughters undergo FGM/C	To stop FGM/C:
		47 % - appearance of external genitalia		81 % - Public enlightenment needed
		27 % - tradition		25 % - Counselling of parents
		11 % - initiation into womanhood		7 % - punishing any person who aids or abets the practice
		7 % - spiritual satisfaction		
Rasheed et al. 2011 [25]	Egypt	100 % - senior physicians believed FGM/C prescribed by religion	Nurses:	NR
		97 % - young physicians believed FGM/C prescribed by religion	88 % - supported the practice of FGM/C	
		88 % - nurses believe it is a traditional practice	48 % - would have their daughters undergo FGM/C	
			28 % - had their daughters undergo FGM/C	
			Young Physicians:	
			34 % - supported the practice of FGM/C	
			Senior physicians:	
			15 % - supported the practice	
Refaat 2009 [26]	Egypt	82 % - do NOT approve of the practice	18 % - supported practice; reasons for continuing practice included:	91 % - FGM/C and complications should be taught at medical school
		Those practising in the Upper Egypt area, those from rural areas and those with a diploma (rather than PhD or Fellowship) were more likely to approve the practice of FGM/C	• Convinced of benefit	40 % believed that physicians are the most appropriate to perform FGM/C
			• Profit	35 % did NOT approve of the law banning FGM/C
			• Harm reduction	
			82 % - did NOT approve of the practice for the following reasons:	
		18 % - supported practice for religious or customary reasons	75 % - reduced sexual pleasure	
			64 % – pain	
			61 % - bad habit	
			52 % - not religious practice	
			49 % - causes health problems	
			48 % - against women’s dignity	
Mostafa et al. 2006 [27]	Egypt	51 % - NO medical reason for performing FGM/C	43 % - unethical for a health professional to damage a healthy body	50 % - medicalization is the first step to prevention of the practice
		45 % - FGM/C is a violation of human rights	65 % - FGM/C is NOT a health issue	23 % - believed that the law is enough for prevention
		34 % - FGM/C is essential part of culture	32 % - would subject their future daughters to this practice	53 % - believe that laws must go hand in hand with community education
		24 % - FGM/C prevents external genitalia from growing	58 % - would NOT object if family members were to subject their daughters to FGM/C	
		20 % FGM/C ensures a girl’s virginity	73 % - FGM/C should be medicalised	
		49 % - prevents promiscuity	91 % - medicalization favourable because it reduces pain; carried out under hygienic conditions and with anaesthetic	
		30 % - FGM/C is a religious obligation		
		86 % - believed that FGMC is practiced only by Muslims		
Onuh et al. 2006 [28]	Nigeria	9 % - decreases promiscuity	4 % - will have their own daughters undergo FGMC	92 % - FGM/C should be legislated against
		10 % - makes genitalia more attractive	3 % - FGM/C is a good practice	
		Other reasons: − cultural; financial; patient safeguarding from “traditional circumcisers”	3 % - will encourage FGM/C	
			24 % - some forms of FGM/C are not harmful	
Publications from “Western Countries”				
Purchase et al. 2013 [30]	UK	76 % - cultural reasons	NR	NR
		16 % - religious reasons		
Relph et al. 2013 [31]	UK	100 % - cultural reasons	9 % - FGM/C should be medicalized to reduce complications	87 % - would warn social services of a child in danger of FGM/C
			18 % - would support a woman’s request for re-infibulation after birth if this was legal in the UK	
Moeed et al. 2012 [20]	Australia and New Zealand	NR	21 % - O&G specialists believed that in the women and girls with FGMC seen by them, the FGM/C was probably done in Australia (but they did not provide number estimates)	NR
			42 % of the FGM/C workers believed that the women and children with FGMC probably had the procedure performed in Australia/NZ	
			26 % of FGMC/C workers believed that children were being taken out of Australia to attend family celebrations and to have FGM/C done overseas	
Kaplan-Marcusan et al. 2009 [33]	Spain	50 % - traditional reasons	NR	2001 -1 % said ignore the problem
		16 % - religious reasons		48 % - educate
				32 % - educate and report
				19 % - report to authorities
				2004 – None said ignore
				49 % - educate and report
				27 % - educate
				24 % - report to authorities
Leye 2008 [34]	Belgium	NR	86 % - FGM/C is a form of violence against women	21 % - believed that FGM/C performed by a medical practitioner would reduce harm
			61 % - FGM/C is a violation of human rights	48 % - wanted more clarity around ethico-legal issues
			7 % - FGM/C should be respected because of cultural and religious beliefs	
			77 % - considered re-infubulation as a form of FGM/C	
			19 % - would re-infibulate if requested by the woman	
			47 % - a symbolic incision was a good alternative to FGM/C	
			15 % - Genital piercings and vaginal cosmetic surgery considered a type of FGM/C	

^a^NR = Not reported

A minority of health professionals practising in high income countries were not against FGM/C. Seven of 344 Belgian doctors felt that FGM/C deserved respect because of cultural and religious connotations [34]. A survey of labour ward health personnel in the UK, showed that 14 % believed that a competent adult should be allowed to consent to FGM/C, 9 % felt that the procedure could be “medicalized” to prevent complications, and 17 % said they would support a woman’s request for re-infibulation [31]. Health professionals from high income countries indicated that they would reluctantly support re-infibulation of women from countries where this is customary to protect the woman from being marginalised from her community [26, 31]. In the ANZ study most respondents believed that it is acceptable to oversew labia majora to prevent infection and fusion, and for patient comfort [20]. Between 15 % and 91 % of Egyptian health professionals surveyed, supported FGM/C if performed by a doctor to minimise harm (Table 5) [25–27].

Health professionals believed that laws will only be effective with the implementation of better awareness and education for patients and the community about FGM/C [24, 33].

## Discussion

Our review confirms that the practice of FGM/C continues and remains prevalent in some African countries despite many having adopted laws against this practice. We found 10 studies confirming that health professionals working in high income countries such as Australia, New Zealand, United Kingdom, Italy, Sweden, Belgium, Spain and Switzerland care for women and girls with FGM/C [4–7, 10–12, 21–23]. Some have been approached to perform FGM/C in babies or young children [20, 24, 34, 37]. Furthermore, health professionals in Australia and New Zealand, the UK, Belgium and Switzerland believed that it was likely that some of their patients with FGM/C had the procedure done in these high income countries despite legislation making FGM/C illegal. Some health professionals did not know about anti-FGM/C laws or were unsure what these laws covered and what their obligations were under the laws [11]. There have been few prosecutions for FGM/C in countries where such laws exist [38]. Laws are not a deterrent if communities perceive that the risk of detection is low and there are few prosecutions [4, 5, 38]. To prevent the practice of FGM/C, health professionals felt that laws were not enough and needed to go hand in hand with awareness campaigns and education for patients and communities, including the men in those communities [24]. This is supported by the recently published UK Multi-Agency Practice Guidelines on Female Genital Mutilation [5].

Our systematic review is limited by the quality of the published studies, many with small sample sizes and low response rates. Although attitudes to FGM/C may differ according to the gender of the health professionals surveyed, this could not be assessed in our review due to inadequate sample description, seven of the 18 studies failing to report the gender of respondents.

The level of knowledge about FGM/C among health professionals varied with most unable to recognise the 4 different types of FGM/C described by the WHO. Few were able to identify countries where FGM/C is prevalent and therefore did not know that women from these countries are at high risk of FGM/C. Health professionals who regularly worked with women from high risk communities and where the health service was targeted to these communities had better knowledge of FGM/C. However, even in a clinic in the UK that sees many women with FGM/C, only 26 % felt that they had adequate training about FGM/C [23].

Only two studies included in our review reported on psychological and psychosocial problems, either immediate or long-term, which are associated with FGM/C [27, 30]. This is consitent with findings from a study by Mulongo et al. and supports the need to raise awareness in health professionals about these under-recognised consequence of FGM/C and the need to provide counselling services to support women and girls affected by FGM/C and their families [8].

Most of the studies surveyed obstetricians, gynaecologists, nurses, midwives and other health professionals working with pregnant women. Only two surveys reported separate data for paediatricians [6, 7]. Paediatricians have an important role in recognising children at risk, preventing FGM/C by counselling parents and communities, reporting children to authorities, and in treating children who have undergone FGM/C and are suffering complications [5, 6, 19]. Of the 18 studies included in this review, only 5 addressed prevention of FGM/C, mainly through counselling women who have FGM/C and have recently given birth, against FGM/C for their daughters [4–6, 10, 11]. This is appropriate as the strongest predictor of a child undergoing FGM/C is the mother having undergone FGM/C herself [5]. However, in a study of Belgian obstetricians and gynaecologists 65 % said they would not undertake to counsel women to prevent FGM/C among their daughters [10]. This may be because they feel inadequately trained and resourced to advocate against FGM/C. In a large survey of Belgian midwives, which was not included in our systematic review as it was only recently published on-line, the majority lacked adequate access to education and guidelines about FGM/C to provide adequate care, and to counsel mothers against FGM/C for their new born daughters [39].

Health professionals need education and guidelines relevant to FGM/C provided both in basic medical training and in continuing medical education. They wanted more information about how to speak with families about this culturally sensitive issue, how to recognise children who might be at risk of FGM/C and how to treat women and girls who have undergone FGM/C. The RACP guidelines on FGM/C provide a short summary of recommendations for paediatricians who may be faced with FGM/C, however, there is no practical guidance of what to do and what to say when dealing with a child with FGM/C or at risk of FGM/C and her family, often within a complex medical and socio-cultural context [40]. Health professionals also called for better education about anti-FGM laws and their obligations under these laws.

As FGM/C often occurs in the community, there is a need for community health workers, general practitioners, community nurses and community paediatricians to be educated about FGM/C and to be provided with clear guidelines about what actions they need to take to prevent FGM/C, including guidance about when and how to report children to child protection authorities. Health professionals must also be provided with appropriate structures within the healthcare system, including referral pathways and specialist services for women and girls with FGM/C, and girls who may be at risk of FGM/C. Such pathways, integrating community prevention with inter-agency, inter-sectoral collaboration including schools, health services and community groups, has been recommended and is being implemented in the UK [5, 19]. Furthermore, healthcare systems, practitioner credentialing bodies and communities have an important role in education and prevention of the medicalization of FGM/C [41].

## Conclusion

This is the first literature review of health professionals’ knowledge, attitudes and practice related to FGM/C. Only 18 studies were identified between the years 2000 and 2014, suggesting that this topic is under-researched. The review highlighted the need for easily accessible educational resources and evidence-based guidelines to enable health professionals to provide culturally sensitive medical and psychological care for women and girls who have undergone FGM/C. Furthermore, health professionals, especially paediatricians and family doctors, need skills to recognise women and girls at risk of FGM/C; they need resources to enable them to counsel girls and their families and communities to prevent this harmful and illegal practice. Most of the research papers reported on obstetricians, gynaecologists and other health professionals dealing with pregnant women. As the immigrant communities in high income countries become larger and increasingly multicultural and ethnically diverse, health professionals are more likely to see women and girls with FGM/C or at risk of FGM/C, in their clinical practice. Further research is needed to determine knowledge gaps and needs for education and resources among other groups of clinicians including paediatricians, general practitioners and community health workers.

## Abbreviations

FGM/C: Female genital mutilation or cutting
PRISMA: Preferred reporting Items for systematic reviews and meta-analyses
